# Improving Healthy Living in Residential Care Facilities: Feasibility, Acceptability, and Appropriateness of Implementing a Multicomponent Intervention for Diabetes Risk Reduction in Adults with Serious Mental Illnesses

**DOI:** 10.1007/s10488-022-01189-z

**Published:** 2022-02-03

**Authors:** David H. Sommerfeld, Amy M. Brunner, Danielle Glorioso, Ellen E. Lee, Cynthia Ibarra, Elizabeth Zunshine, Rebecca E. Daly, Christine Zoumas, Dilip V. Jeste

**Affiliations:** 1 Department of Psychiatry, University of California San Diego, 9500 Gilman Drive, La Jolla, CA 92093 USA; 2 Sam and Rose Stein Institute for Research on Aging, University of California San Diego, La Jolla, CA 92093 USA; 3 VA San Diego Healthcare System, San Diego, CA 92161 USA; 4 Moores Cancer Center, University of California San Diego, La Jolla, CA 92037 USA; 5 Department of Neurosciences, University of California San Diego, La Jolla, CA 92093 USA

**Keywords:** Schizophrenia, Obesity, Diet, Exercise, Smoking

## Abstract

Persons with serious mental illnesses experience high rates of medical comorbidity, especially diabetes. This study examined initial implementation feasibility, acceptability, and appropriateness of a new 6-month Multicomponent Intervention for Diabetes risk reduction in Adults with Serious mental illnesses (MIDAS) among persons in residential care facilities (RCFs). We conducted a mixed-methods study using four types of quantitative and qualitative data sources (administrative data; structured facility-level observations; resident assessments including blood-based biomarkers, 24-h dietary recalls, and self-report physical activity; and focus groups/interviews with staff and participants), to assess evidence of and factors affecting intervention feasibility, acceptability, and appropriateness. It was feasible to provide a high percentage of MIDAS class sessions (mean 50 of 52 intended sessions delivered) and make nutrition-related RCF changes (substitutions for healthier food items and reduced portion sizes). Class attendance rates and positive feedback from residents and staff provided evidence of MIDAS acceptability and appropriateness for addressing identified health needs. The residents who attended ≥ 85% of the sessions had greater improvement in several desired outcomes compared to others. Implementing a fully integrated MIDAS model with more extensive changes to facilities and more fundamental health changes among residents was more challenging. While the study found evidence to support feasibility, acceptability, and appropriateness of individual MIDAS components, some challenges for full implementation and success in obtaining immediate health benefits were also apparent. The study results highlight the need for improving health among RCF populations and will inform MIDAS adaptations designed to improve intervention fit and effectiveness outcomes.

## Introduction

Persons with schizophrenia and other serious mental illnesses (SMIs) such as bipolar and schizoaffective disorders often experience physical health conditions such as cardiovascular diseases which contribute to a 2–12 times higher mortality rate than age-comparable individuals from the general population (Brown, 1997; Casey et al., 2009; Chesney et al., 2014; Crump et al., 2013; Olfson et al., 2015; Piatt et al., 2010; Tsuang & Woolson, 1978). A recent review of the literature showed that the mortality gap between people with schizophrenia and the general population has increased in recent decades due to health behaviors (e.g., higher rates of smoking and limited physical activity) and poor access to physical health care for persons with SMI compared to the rest of the population (Lee et al., 2016). There is growing evidence that age-associated physiological changes occur earlier in persons with SMI (Beary et al., 2012; Bobes et al., 2010; Jeste et al., 2011; Kirkpatrick et al., 2008; Vancampfort et al., 2011), with specific studies strongly supporting the hypothesis of accelerated biological aging in persons with schizophrenia (Hong et al., 2016; Joseph et al., 2015; Kirkpatrick et al., 2008; Koutsouleris et al., 2014; Lee et al., 2016; Wright et al., 2014). Overall, the most common cause of excess mortality in persons with SMI is cardiovascular disease (Crump et al., 2013; Olfson et al., 2015; Piatt et al., 2010), for which diabetes is a major risk factor.

Individuals with SMI may have a higher genetic predisposition specifically to type 2 diabetes (Gough & O’Donovan, 2005; Ward & Druss, 2015). Other risk factors for diabetes in SMI include: (1) lack of knowledge about diabetes self-management (Blixen et al., 2016; Sajatovic et al., 2016), (2) unhealthy lifestyle (energy dense or obesogenic diet, lack of physical activity, sedentary behavior) (Hennekens, 2007; Hert et al., 2009; McKibbin et al., 2006), (3) high body mass index (BMI), visceral obesity, and hyperlipidemia (Chouinard et al., 2016; Joseph et al., 2015; Mitchell et al., 2013), (4) cigarette smoking (Buckley et al., 2010; Weinberger & George, 2010), (5) chronic exposure to antipsychotic medications, which are associated with weight gain, dyslipidemia, cardiovascular disease, and decreased glucose tolerance (American Diabetes Association et al., 2004; Correll et al., 2014; Galling et al., 2016; Heald, 2010), (6) poor access to and coordination of primary healthcare, exacerbated by poverty (Blixen et al., 2016), and (7) psychosocial stress (Cimo et al., 2012). Given that several of these factors are potentially modifiable or at least manageable, Hoang et al. have called this “avoidable mortality” in people with SMI (Hoang et al., 2013).

Multiple strategies have been developed to address physical health concerns among persons with SMI including the use health coaches to reduce obesity (Bartels et al., 2015), utilization of peers to provide health-related supports (Cabassa et al., 2017), and coordinated health coaching, education, and mental health care to achieve health goals (Daumit et al., 2020). However, limited research explicitly focuses on the population of persons with SMI who live in residential care facilities (RCF). One such intervention, the Diabetes Awareness and Rehabilitation Training (DART) provided health education services to patients living in residential care facilities (RCFs) who were also attending day treatment programs (McKibbin et al., 2006, 2010). Persons living in RCFs represent a unique population of interest as they must typically be substantially impaired as a pre-condition for becoming eligible to reside in an RCF, but for whom their living environment is potentially more conducive to structured changes to promote healthy living given that meals, activities, and social supports are all located where the person resides.

Accordingly, we developed a new Multicomponent Intervention for Diabetes Risk Reduction in Adults with Serious Mental Illness (MIDAS) by building upon the successes of DART. MIDAS was designed for adults of all ages with SMI who live in RCFs and who have diabetes or are at a risk of developing diabetes. Torrey (1995) estimated that one-sixth of the people with SMI may be residing in RCFs, and the proportion of people with SMI living in RCFs is expected to grow as the number living with families continues to decline (Cabassa et al., 2015; Craig & Lin, 1981; Goldman, 1982; Solari et al., 2014; Somers et al., 2017; Tsai et al., 2011). RCFs provide a fairly stable form of housing with 78% of residents living in an RCF for more than 13 months (Ireys et al., 2006), allowing enough time for residents to participate in in-house, multi-session classes over the course of several months.

The underlying conceptual model for MIDAS is that educating RCF staff and residents about the why, what, and how of lifestyle changes necessary to manage and prevent diabetes and facilitating their implementation would support longer-lasting behavior changes by increasing participants’ knowledge and self-efficacy. Facility activity directors (ADs) were provided training and materials to teach wellness classes that educated their residents about making healthy decisions regarding eating, exercising, and smoking reduction. In addition, since the majority of meals are eaten by residents on-site, the MIDAS also included working with the RCF’s kitchen staff on making healthy dietary changes to the food they provided.

Following the nomenclature articulated by Curran et al. (2012), the overall MIDAS study was designed as a type 1 hybrid effectiveness-implementation study with the primary emphasis on assessing intervention effectiveness. For this research, we focus specifically on the MIDAS implementation context as this represents a critical factor affecting evidence-based practice (EBP) implementation and sustainment (Aarons et al., 2011; Greenhalgh et al., 2004; Klein & Sorra, 1996). Given that intervention “fit” is typically comprised of both structural/practical and value-based/ideological aspects (Aarons et al., 2011), this study examines three specific domains of fit, related to both RCF residents and staff, as key implementation outcome indicators: (1) intervention feasibility, (2) acceptability, and (3) appropriateness (Proctor et al., 2011; Weiner et al., 2017). Feasibility focuses on capabilities of the staff and work-flow processes to assess the extent in which it is possible/realistic to implement key components of an intervention. Acceptability is also related to intervention fit, but has a more personal value-based dimension that focuses on the extent to which there is a desire or interest in making the recommended service changes. Thus, an intervention could be perceived as feasible to implement (i.e., technically capable), but not desirable enough by front-line staff or organizational leadership to be considered acceptable to implement, or vice versa. Thirdly, an intervention needs to be considered appropriate or relevant to achieving an agreed upon objective.

## Methods

### Participants

The study protocol was reviewed and approved for the ethical treatment of human subjects by the Institutional Review Board (IRB) of University of California San Diego. All persons with schizophrenia or schizoaffective disorder living in the RCFs were informed of the MIDAS study and invited to participate through mailings, flyers, and individual interactions with MIDAS research team members. All the RCF staff and residents participating in the MIDAS study provided a written informed consent.

The Substance Abuse and Mental Health Services Administration (SAMHSA) criteria for RCFs (known in California as Board-and-Care Homes and as group homes or family care homes in other states) include specialization in serving persons with SMI, provision of 3 meals/day, 24-h staff supervision, stay of > 30 days, and assistance with medications, hygiene, and transportation (Ireys et al., 2006). In California, licensed RCFs that have Augmented Services Program (ASP) contracts with their county and a full-time Activity Director (AD) receive financial support for services, education, employee incentives. An AD must have more than 2 years of experience working with people with SMI and they must offer two activities per day.

This study focused on the MIDAS implementation experiences and outcomes at the first three RCFs for adults with SMI in our trial. A total of 44 residents therein enrolled in MIDAS and completed baseline assessments. These residents’ mean age was 48.7 years (range 26–65); 55% identified as males and 45% as females. The residents represented a racially diverse group with 43% identifying as Caucasian, 23% as African American, 23% as Hispanic, 5% as Asian American, and 6% as multi-racial. Almost all (95%) were single. For RCF staff, the average age was 46 years, with 86% identifying as females; 71% as Hispanic and 29% as Asian American.

A total of 37 residents participated in at least one of the MIDAS class sessions with 23 (62.1%) participating in one of the three resident-only focus groups (i.e., one conducted at each RCF with all MIDAS participants invited to attend the focus group). Three additional interviews/group interviews (one at each RCF) were conducted with RCF ADs (n = 3; 100% of RCF ADs), and RCF managers (n = 2; 66.7% of RCF managers). One of the interviewed ADs was also the RCF cook and a separate group interview was conducted with the primary cooks for the other two RCFs (n = 2). The supplemental cooks who covered select meals or filled-in for absences did not participate in the interviews. The focus groups and interviews lasted 30–60 min and were conducted on site at each RCF between January and March 2020 following the conclusion of the intervention.

### MIDAS

This is a manualized intervention, and the manual was taught and provided to the core RCF staff (AD, cook, and manager). The staff attended an 8-h training session prior to the intervention initiation. The research dietitian provided follow-up training for the RCF cook, regarding use of standardized portions and menu substitutions to improve nutrition. The main components of the intervention were as follows.

(I) *Group education* There were two sessions, each for 20–40 min, every week over 26 weeks, for a total of 52 sessions, led by the AD. Themes that were repeated throughout the intervention included: General health knowledge (education about diabetes risk, obesity, and goal setting), Nutrition (education regarding food groups and portions, meal planning, and eating for special occasions), Physical activity (exercise benefits, activity tracking, and overcoming activity barriers) and Smoking Cessation/reduction. While the resident education components of MIDAS had similarities with the substantive content of other diabetes self-management programs (e.g., promoting healthy eating and increased physical activity), given the desire to appeal to and be relevant to as many residents in an RCF as possible (i.e., both those with and without an existing diabetes diagnosis), MIDAS contained less emphasis on diabetes-specific assessment practices such as regular blood sugar monitoring.
(II) *Nutritional intervention* The registered research dietitian met with the RCF manager and cook to evaluate the current meal plan and suggested possible menu and ingredient changes that were cost-effective and focused on portion and calorie control, nutrient density, and dietary guidelines for diabetes prevention. These took into consideration current menu options and the facility’s dietary patterns by making small changes to existing recipes, such as decreasing high-energy dense ingredients and increasing low-energy dense ingredients.
(III) *Group physical activity routines* The aim was to increase participants’ physical activity through education, guided walks, and exercise routines. As part of the twice-weekly groups, participants took part in short (15-min) guided walks in local community or a low-intensity physical activity routine. Subjects who exercised longer than 10 min/day were instructed to aim for an incrementally greater goal (e.g., weekly increases of 5 min more per day until a goal of 60 min/day is reached) and to plan to increase intensity as tolerated. The sessions also focused on reducing sedentary behaviors, given their association with metabolic dysfunction.
(IV) *Smoking cessation/reduction* We used manualized content consistent with recommendations of the Clinical Practice Guideline for treating Tobacco Dependence (Fiore et al., 2008)

Each resident was also provided a workbook with key session information and activities/homework to further engagement with the course content. Initially there were issues with residents forgetting or misplacing their workbooks, so a change was made to have the ADs collect the workbooks at the end of each session. With this shift, homework activities were completed in the workbooks, collected at the end of the session and then revisited and discussed at the start of the next session.

### Data Sources and Analysis

Four data sources were utilized to assess MIDAS feasibility, acceptability, and appropriateness as follows.

1. The administrative data were comprised of MIDAS session attendance logs that indicated the dates of session delivery and the residents who participated in each session. The attendance logs from each RCF were reviewed to generate total number of sessions provided by each RCF (comparing that number to the expected MIDAS intervention duration of 52 sessions). This MIDAS session provision value was calculated for each RCF and the overall cohort average was used as an indicator of the feasibility of consistently providing MIDAS sessions at RCFs. To assess MIDAS session acceptability, the average participation rate (i.e., total number of MIDAS sessions attended by participants divided by the total number of participants attending any class sessions) was calculated for each RCF and the overall RCF cohort.
2. The structured observations of meal preparation and provision were completed by our registered dietitians. These observational visits were not completely unannounced, as typically there was 1 day or less notice provided to the RCFs prior to the actual visit. The structured observations covered many different domains such as the availability of certain food and beverage items (e.g., fruits and vegetables, sugary drinks, snacks, fresh water), portion control mechanisms (e.g., use of serving utensils, availability of “seconds”), and visibility of signage promoting healthy eating and drinking. We compared initial structured facility observations with the final observations to identify changes in how food or beverages were prepared and/or provided to residents. For example, the added sugar component of the observational assessment examined whether, at follow-up, there were: (1) more low sugar beverages available, (2) sugar substitutes and/or limits on added sugar for coffee/cereal, and (3) more low-sugar and then high-sugar cereals available. These items were scored dichotomously as 0 or 1 corresponding to “no” or “yes”.
3. Resident assessments of five key indicators of healthy living were obtained immediately before starting and at the end of the intervention. These included body mass index (BMI), hemoglobin A1c, grams of added sugar intake per day, percentage of calories from fat, and the International Physical Activity Questionnaire (IPAQ; Lee et al., 2011), a four-item measure of self-reported physical activity over the last seven days. Added sugar intake and the calories from fat were assessed via comprehensive 3-day dietary recalls completed by the MIDAS team with residents every three months and analyzed with the Nutrition Data System for Research (NDSR). Descriptive statistics (i.e., means and standard deviations) from the pre-MIDAS key indicators of healthy living were used to characterize RCF resident health and healthy living behaviors at baseline. These initial values were compared to those at the conclusion of the six-month intervention to assess signals of healthy living improvements in 33 residents who had data from both time points.
4. Seven RCF focus groups and interviews were conducted with MIDAS stakeholders to obtain feedback about experiences from RCF residents and staff. The notes from each of these sessions were entered into the Dedoose qualitative data analysis software package (Dedoose8.3.45, 2021). To conduct our analysis, we began by open coding of the notes to identify text and themes relevant to understanding stakeholder perceptions of MIDAS within the broad domains of feasibility, acceptability, and appropriateness (Corbin & Strauss, 2008). Two investigators independently coded each set of focus group/interview notes to identify: (1) evidence of MIDAS feasibility, acceptability, and appropriateness (including aspects considered not to be feasible, acceptable, or appropriate), and (2) factors affecting MIDAS feasibility and acceptability. Where discrepancies existed, the investigators discussed the rationale for each code and arrived at a consensus code by creating a new code or agreeing to use an existing one. Coded text excerpts were then reviewed to identify core themes related to understanding MIDAS feasibility, acceptability, and appropriateness. To further assess the validity of the qualitative findings, a preliminary summary of the themes from the qualitative data were reviewed by two additional MIDAS team members who had regular interactions with RCF residents, cooks, and ADs. These team member reported that the findings were consistent with their experiences and feedback they had received.

Using Palinkas et al. (2011) mixed-method nomenclature, this study is QUAL + QUANT, with a primary objective of examining data convergence in that the multiple forms of data were: (1) analyzed simultaneously, (2) of equal importance, and (3) both intended to contribute to the overall understanding of MIDAS feasibility, acceptability, and appropriateness, even though the specific focus of the different types of data varied to some extent. The use of the qualitative data to conduct a detailed examination of the factors affecting MIDAS feasibility and acceptability also reflected a complementarity perspective in that the qualitative data were able to answer questions not addressed via the quantitative data.

## Results

In the following sections we present the mixed-method results related to MIDAS feasibility, acceptability, and appropriateness. For MIDAS feasibility and acceptability we also include findings related to the key factors identified as affecting feasibility and acceptability.

### MIDAS Appropriateness

#### Intervention Appropriateness: Focus Groups/Interviews

Residents indicated that a primary reason they agreed to participate in MIDAS was that they were aware of risks of diabetes, being overweight, and smoking, and were interested in healthy living. Likewise, RCF ADs, managers, and cooks acknowledged the need for improving resident physical health. As one staff member said, “We care for our residents, and we want them to be healthy”. MIDAS was viewed as appropriate for addressing physical health needs identified by both residents and RCF staff. Participants and staff indicated areas where they felt they had seen positive changes—walking more, feeling less stressed, having more energy, losing weight, and learning skills that they thought were appropriate for achieving their goals.

#### Intervention Appropriateness: Resident Assessments

We examined pre- and post-MIDAS assessments separately for residents who attended at least 85% of the sessions (i.e., 44 of 52) and those who attended less than 85% of the sessions. Resident assessments prior to starting MIDAS suggested a need for improved health among both groups of residents (Table 1). Baseline hemoglobin A1c levels were particularly elevated (6.7%) among those who attended a high number of MIDAS sessions and for both groups of residents, all other assessments of BMI, added sugars, percent of calories from fat, and physical activity indicated that on average MIDAS participants were obese, had unhealthy dietary behaviors, and exhibited low levels of physical activity before starting MIDAS.

**Table 1:** Key indicators of resident healthy living in more adherent versus less adherent residents

Indicator of healthy living	Attended < 85% of MIDAS sessions (n = 17)			Attended at least 85% of MIDAS sessions (n = 16)		
	Pre-MIDAS Mean (S.D.)	Post-MIDAS Mean (S.D.)	Cohen’s d effect size	Pre-MIDAS Mean (S.D.)	Post-MIDAS Mean (S.D.)	Cohen’s d effect size
Hemoglobin A1c	5.7 (0.5)	5.6 (0.7)	0.6	6.7 (2.1)	6.3 (1.7)	0.5
Body mass index (BMI)	36.7 (7.4)	36.3 (6.8)	0.2	32.0 (9.3)	31.7 (8.5)	0.1
Percent of daily calories from fat	35.4 (5.9)	40.2 (6.5)	− 0.7	36.2 (6.1)	38.2 (3.2)	− 0.4
Added sugars (grams)	108.9 (61.2)	106.7 (61.2)	0.1	119.6 (80.0)	96.2 (41.4)	0.3
Physical activity (1 = low activity and 3 = high activity)	1.4 (0.5)	1.1 (0.3)	− 0.5	1.2 (0.6)	1.4 (0.5)	0.2

Negative sign for Cohen’s d indicates change in undesired direction

While small sample sizes precluded statistical testing, a descriptive examination of residents’ assessments following the 6-month MIDAS intervention showed mixed signals of change. For all residents there were signals of a reduction in A1c levels (estimated as a “medium” effect size for both). The reduction was particularly pronounced among those who attended a high number of MIDAS sessions with the average A1c level lowering from 6.7 to 6.3%, which is substantially closer to the A1c level of 5.7% or less recommended by the American Diabetes Association (2021). For both resident groups, mean BMI was essentially unchanged while the percentage of calories from fat increased at the conclusion of MIDAS. Among residents who participated in at least 85% of the MIDAS sessions, the mean number of grams of added sugars dropped by approximately 20%, although still remaining well above the recommended levels of ≤ 48 g per day for a 2000 cal per day diet. Physical activity increased slightly for those who had high levels of MIDAS participation, but decreased among those who did not participate as regularly in MIDAS sessions.

### MIDAS Feasibility

#### Implementation Feasibility: Administrative MIDAS Class Data

Administrative data on participation of residents in the MIDAS sessions showed that 96% of intended/planned MIDAS sessions (i.e., 50/52 MIDAS sessions) were provided across the three RCFs, indicating a high degree of feasibility.

#### Implementation Feasibility: Facility Observations

Structured observations identified three primary types of facility-level changes in the RCFs at the end of the intervention: (1) Signs (provided by the UCSD MIDAS team) were posted in common areas in the RCFs that promoted drinking more water, learning about sugar content in common drinks, and being aware of the impact of too much sugar in health conditions such as diabetes, heart and liver diseases. (2) RCFs installed filtered water machines to encourage residents to drink fresh water instead of sugary drinks and increased the amount of fruits and vegetables they were providing as part of residential meals and snacks. (3) Observable reductions in unhealthy/less healthy food and beverages were achieved via changing practices and changing materials/equipment. Also, the RCF cooking staff implemented several new practices from limiting “second helpings” on food to reducing the amount of sugar used to make drinks or butter used in preparing food to using smaller portioned serving utensils, plates, bowls, cups, and new condiment dispensers (provided by the UCSD MIDAS team) to standardize portions to a healthier size.

#### Implementation Feasibility: Focus Groups/Interviews

Cooks indicated that they encouraged residents to read the signs as reminders of the need to be healthy and to help justify some of the facility-level changes (e.g., reduced sugar in drinks) they were implementing. Cooks also tried to incorporate more nutritious food items (e.g., vegetables) into existing menu items, making snacks more healthy (e.g., offering whole fruit and blended “smoothie” drinks), and more frequent provision of healthy sides with meals such as salads. Finally, they reduced portion sizes through the use of the new plates and glasses and reducing the number of high calorie snacks provided to residents—e.g., putting two, instead of four, cookies in each snack bag for residents.

#### Factors Affecting Feasibility of MIDAS Class Participation

Participants and ADs both generally agreed that offering MIDAS classes twice a week was an appropriate frequency. The classes typically lasted between 20 and 40 min, which was considered optimal by ADs who commented that some residents had relatively short attention spans that made it difficult to focus for longer periods of time.

Regarding knowledge gained from the classes, participants specified learning about counting calories, reading nutrition labels, establishing healthy portion sizes, reducing sugar intake, and health benefits of good nutrition and exercise. Several participants commented that they learned more about their diabetes and how to take better care of themselves. A favorite lesson mentioned by multiple residents involved the residents reviewing a menu from a fast-food restaurant and discussing what they would typically order and then looking to find something else on the menu that would be a healthier alternative to their ‘normal’ menu choice.

At the same time, some residents found parts of the material taught to be challenging and difficult to comprehend. Such comments were often, but not always, followed up by an acknowledgement of the important assistance provided by the ADs. As expressed by one resident “When I saw the material I thought how can this be different? It was very different! Mutual respect. Very fair. Not labeling us as mental health patients. They presented the material in a way that was beneficial”.

Overall, the feedback suggested that the material was detailed enough to not solely reflect “common sense”, but structured in a manner that was generally feasible for residents to engage with the course material, particularly when supported by an attentive class leader who clarified areas of potential confusion.

#### Factors Affecting Feasibility of MIDAS Class Provision

Focus group discussions with ADs and managers revealed that they often had multiple responsibilities and helped support other tasks within the facility (e.g., housekeeping). Despite their busy schedules, the 20–40 min classes twice a week were feasible, though the ADs often did not have time to review the class material in detail prior to conducting the sessions.

While ADs commented that it would be ideal to have more time to prepare for the sessions, they all spoke very favorably of the structured MIDAS course materials and the instruction manual that guided them through the activities and course content for each session.

ADs indicated that the training and support received from MIDAS research team was helpful, and they also felt supported by the UCSD trainers during the intervention. The ADs who spoke English as a second language expressed that they did not always feel comfortable with their ability to communicate all the session content to residents. To address this concern, UCSD staff helped co-lead sessions as needed to explain material that was challenging for the ADs to communicate. This was appreciated by ADs.

#### Factors Affecting Feasibility of MIDAS Facility-Level Nutrition Changes

Focus group feedback with cooks and managers indicated three primary factors that facilitated an RCF’s ability to make recommended changes: (1) MIDAS training/coaching; (2) obtaining new materials/supplies (e.g., plates and cups) to support changes, and (3) emphasizing low burden/low cost change options. The cooks felt supported by the MIDAS team. As noted above, however, the MIDAS team did not work directly with all cook staff personnel who may provide some meals at a given facility, which contributed to some disruption in implementation of facility-level changes. The provision of moderately sized plates and glasses, serving scoops, etc., was viewed very positively by the cooks. Given the busy workflow for kitchen staff and the need to not make increase costs, changes were generally targeted toward factors that could be enacted relatively easily without additional expenses (e.g., reducing availability of sugary cereals, adding less sugar to food and drinks). The cooks stated that more substantial changes like adding completely new healthy meals were not considered feasible.

### MIDAS Acceptability

#### Resident Acceptability: Administrative MIDAS Class Data

Across all 37 residents who participated in MIDAS classes, the mean number of sessions attended was 34 (65% of all planned sessions). For the first 26 sessions, the mean number of sessions attended was 19 (73% of planned sessions), whereas for the last 26 sessions (i.e., second time through the MIDAS course material), the mean number of sessions attended was 15 (58% planned sessions). There was some reduction in attendance over time, but a substantial group of residents (43%) maintained high rates of participation throughout, by attending at least 85% of all sessions (44/52 sessions).

#### Resident Acceptability: Focus Groups/Interviews

Residents generally found the MIDAS sessions interesting and enjoyable (discussed in more detail below in the section examining factors affecting MIDAS acceptability) and at least some MIDAS participants were more aware of what they were eating and sought to make healthier decisions, particularly related to reducing sugar intake. The cooks provided a more nuanced perspective regarding the acceptability of the various facility-level changes to provide healthier food and beverages. They found that some residents were not pleased with the changes (e.g., reduced portions, less sugar in coffee, etc.) at first, but over time they were more accepting of the changes as they became part of their normal routine. However, not all of the cooking staff (e.g., those on evening shifts) received the MIDAS training and did not adhere to the healthier approach to providing food. This difference in food provision by different cooking staff would sometimes lead to resistance from residents in transitioning back to the healthier foods of the MIDAS-trained cooks.

#### Factors Affecting Acceptability of MIDAS Class Attendance

Most comments related to the course content had a positive orientation and focused on the things they learned, and generally found the classes interesting. As one resident said, the classes were “not boring at all”. Participants said that they enjoyed the social interactions fostered by the group discussions and that they helped each other learn the material. Another factor that encouraged class attendance identified by the participants was the provision of healthy snacks and drinks at the class as well as nominal financial incentives (i.e., $1 per class distributed via $10 gift card after attending 10 sessions). One AD indicated their belief that some MIDAS participants might have been motivated to attend the classes primarily due to the incentives, whereas others were there because they really wanted to learn the material.

#### Factors Affecting Acceptability of MIDAS Class Provision

All ADs spoke favorably of the MIDAS classes and expressed support for the goal of improving the residents’ health. The ADs felt they had learned new health information while leading the MIDAS classes and were trying to lead healthier lives themselves. They also commented on their positive interactions with the residents during the classes.

#### Factors Affecting Acceptability of MIDAS Facility-Level Nutrition Changes

The cook staff commented that they believed they could see positive changes with at least some residents as having more energy and potentially losing weight. The cooks were highly supportive of the changes recommended if there were minimal time or effort implementation burdens.

### Summary of Mixed-Method MIDAS Feasibility, Acceptability, and Appropriateness Results

As seen in Table 2, the pattern of findings indicated a high degree of convergence between the qualitative focus group/interview data and the other forms of data examined in this study. Thus, key evidence of implementation feasibility identified in both the facility observations and the focus groups/interviews included awareness of the new signage at the RCFs provided by MIDAS that promoted healthy living, efforts to implement healthier food and beverage substitutions, and an emphasis on reducing portions. Likewise, MIDAS was considered to be appropriate for RCF residents (i.e., high need for health improvements among RCF residents and some signals of achieving desired health improvements). The attendance data showed high levels of consistent session attendance, and feedback from the focus groups indicated that residents found the sessions interesting and enjoyable suggesting high levels of intervention acceptability. Table 3 provides an overview of the key factors (discussed above) that affected MIDAS feasibility and acceptability.

**Table 2:** Summary of evidence indicating MIDAS feasibility, acceptability, and appropriateness

Data source	Implementation feasibility	Resident acceptability	Intervention appropriateness
Admin. data	1. Consistent class provision	1. Regular class attendance	Not assessed with this data source
Facility observation	1. Healthy nutrition signage 2. Food/beverage substitutions 3. Reduced food/beverage portions	Not assessed with this data source	Not assessed with this data source
Interviews/focus groups	1. Healthy nutrition signage 2. Food/beverage substitutions 3. Reduced food/beverage portions	1. Residents enjoyed the class sessions and found them interesting 2. Varied resident responses to nutrition changes	1. Perceived need for residents to improve health 2. Perception that some residents’ health improved due to MIDAS
Resident assessments	Not assessed with this data source	Not assessed with this data source	1. Assessments indicated need for improvements in healthy living 2. Mixed initial improvement results

**Table 3:** Summary of key factors affecting MIDAS feasibility and acceptability

Intervention component	Primary group	Feasibility (i.e., able to accomplish)	Acceptability (i.e., want to accomplish)
Classes	Residents	1. Frequency/duration (+) 2. Difficulty level (+/−)	1. Interesting content (+) 2. Social interactions (+) 3. Incentives (+)
	Activity director	1. Frequency/duration (+) 2. Materials (+) 3. Training/support (+) 4. Language barriers (−)	1. Interesting content (+) 2. Positive resident/leader relationships (+)
Facility-level changes	Cooks/manager	1. Training support (+) 2. Difficulties training all relevant cook staff (−) 3. Low burden/costs (+) 4. New materials/supplies (+)	1. Desirability of nutrition related facility changes (+/−)

“+” indicates a facilitating factor and “−” indicates an inhibiting factor

## Discussion

Overall findings from the mixed-method analyses indicated that implementing the individual components of MIDAS in RCFs was generally considered feasible, acceptable, and appropriate by both residents and staff, although with some caveats. Consistent with research identifying a high prevalence of physical health concerns, such as cardiovascular disease and factors promoting diabetes among persons with SMI (e.g., Crump et al., 2013; Olfson et al., 2015), the baseline resident assessment data indicated a clear need for health improvements among RCF residents based on high BMI, limited physical activity, and unhealthy dietary practices. Initial exploratory examinations of MIDAS effectiveness demonstrated some areas of improvement, particularly among those with ≥ 85% attendance at MIDAS sessions, including reductions in hemoglobin A1c, added sugars, and an increase in physical activity. These findings were consistent with feedback from the focus groups and supported the perception among both residents and staff that MIDAS was appropriate for improving health among RCF residents. Regarding individual components of the MIDAS intervention, administrative data demonstrated that MIDAS classes could be successfully provided twice per week over the course of 26 weeks; however, feedback from the focus groups indicated that ADs for whom English was a second language and who may not be fully comfortable with their English speaking abilities may have challenges delivering the intervention. This issue was addressed in the current study by the UCSD MIDAS team providing co-leading assistance as needed to help with language challenges.

A sizable minority of residents (43%) attended ≥ 85% of the 52 total sessions, suggesting their capacity and interest in engaging in longer-term health education activities. The mixture of easy accessibility (i.e., onsite programming), interesting course content, positive social interactions with peers and class leaders, and modest incentives for class participation were thought to be helpful. This finding is consistent with other studies of multi-session/multi-month health promotion interventions for persons with SMI. For example, an intervention that included 18-months of health coaching sessions had a median participation rate that corresponded to 74.5% of total health coaching sessions (Daumit et al., 2020). These findings indicate that while some intervention dropout is to be expected over time, many persons with SMI are capable of maintaining participation in multi-month/multi-session type interventions, particularly when there is an emphasis on reducing barriers to participation (e.g., having onsite classes) and attending to motivational needs (e.g., providing information that is interesting and relevant to their personal goals, offering small participation incentives, etc.).

A unique contribution of MIDAS was the effort to improve the health of persons with SMI by creating positive environmental changes regarding the types and quantity of food provided to residents given that most similar interventions rely primarily on behavioral, pharmacological, or clinical approaches (McGinty et al., 2016). The preliminary outcomes for MIDAS suggest that an environmental strategy is worth pursuing, where feasible, in other SMI-oriented health promotion interventions since desired changes in types and amount of food and beverages provided, brought about through individualized coaching by our nutrition specialists, were usually feasible, if they did not substantially increase time or cost burden. One challenge specific to MIDAS implementation was that not all the cook staff who provided meals at the RCFs participated in the MIDAS coaching. The resultant variability between cooks created some tension as some residents seemed to resist returning to the healthier approaches. Promoting greater awareness of MIDAS facility-level nutrition recommendations and “buy-in” among all cook staff who serve meals at the RCFs is necessary. In this regard, other health improvement interventions may want to incorporate more of an environmental perspective to identify potential malleable changes such as food provision at other relevant organizations serving SMI populations (e.g., adult day care centers or clubhouses).

While the findings provide evidence for feasibility, acceptability, and appropriateness of the MIDAS intervention components in RCFs, we also identified challenges with getting all MIDAS components simultaneously implemented and integrated at each RCF. These difficulties may have contributed to the limited effectiveness outcomes evident in the resident assessment data. Variations in RCF staffing characteristics, particularly confidence in English language skills, as well as openness to engage in more substantial nutrition changes like switching to healthier menus inhibited complete implementation of MIDAS. These observations suggest opportunities for improving the fit of MIDAS with the “real world” RCF environment.

Efforts to improve intervention fit typically focus on adapting the intervention and/or adapting the organizational context (Aarons et al., 2011). For future iterations of implementing MIDAS within RCFs, we intend to utilize both strategies. There is a need for simplifying the course content (i.e., less didactic material) and making it more activity- and group discussion-based. Additionally, an activity and discussion-oriented approach to session delivery, with greater attention to the application of the material to daily living, should help with maintaining resident engagement and minimize drop out.

To change the organizational environment, we need to devote greater attention to generating MIDAS intervention “buy-in” among all the relevant staff members at each RCF. The extent to which key organizational stakeholders such as leaders, supervisors, and frontline staff demonstrate commitment to a particular organizational change has significant implications for the degree to which a change initiative will be successful (Greenhalgh et al., 2004). As indicated by our focus groups, disruptions in the provision of healthier food and beverages due to instances where non-MIDAS coached RCF cook staff were providing meals created stress for some residents and other cook staff. In future MIDAS implementation, the MIDAS nutrition team will expand their education and outreach activities to include communication with the broader group of cook staff who is responsible for some RCF meals. We will also encourage RCF owners and managers to demonstrate more visible and frequent support by rewarding effective staff members.

While this round of MIDAS implementation was not affected by the onset of the COVID-19 pandemic during 2020, since then, many RCFs restricted outsider access to only essential staff onsite as part of their efforts to protect residents. These experiences highlight the importance of planning for future MIDAS implementations efforts that involve minimal or no onsite presence of the MIDAS team and relying more heavily on technology (e.g., telephone and video conferencing) to maintain contact with and communicate core MIDAS objectives to RCF staff. Adapting MIDAS to be more easily deployed using technology (and limited onsite interactions) is consistent with the primary lessons learned from the current study, which demonstrated overall feasibility and acceptability of MIDAS intervention while still identifying needs for simplified and consistent information for all RCF staff.

This study has several limitations. It was a small scale mixed-methods study whose results have unknown generalizability to all RCFs. Additionally, approximately one-third of all RCF residents did not participate in the focus groups and they may have had differing views about their MIDAS experiences. Nonetheless, similar implementation challenges were experienced across the three sites and many of the findings were consistent with known implementation facilitators and barriers. Thus, the circumstances affecting implementation of MIDAS that occurred in these facilities may be relevant to other RCFs as well. Finally, while the small sample size precludes definitive assessment of MIDAS quantitative effectiveness outcomes, pairing preliminary investigations of key anticipated changes with examinations of implementation processes represents an important step in the research process to increase the likelihood of achieving desired outcomes within complex, multi-dimensional interventions such as MIDAS. This type of initial mixed-method assessment can help identify where adjustments to intervention and/or implementation strategies may be needed to fit contextual realities.

The findings from this study highlight the fact that RCFs, with adequate support (i.e., training and coaching), and “buy-in” from RCF residents and staff members, represent feasible settings for implementing interventions to improve lives of persons with SMI. Additionally, this study demonstrates the viability of maintaining participation in multi-month health promotion classes with an extensive number of sessions (over 50) among persons with SMI when classes are offered at their place of residence. The findings also reinforce a need for strategies to support healthy living among RCF residents as various assessments indicated concerns about physical health conditions, diet, and exercise.

From a methodological perspective, the study findings provide further evidence of the benefits associated with conducting hybrid implementation and effectiveness research (Curran et al., 2012) and utilizing multiple data sources from which to examine outcomes and implementation processes (Palinkas et al., 2011). By applying these approaches early on in the research process we were able to identify both implementation successes and limitations that informed adaptions to the MIDAS intervention. It is expected that such adaptations will result in improved effectiveness. This type of research with an emphasis on implementation factors is consistent with recommendations from a recent comprehensive review of intervention studies to address medical conditions among persons with SMI (McGinty et al., 2016), given the importance of identifying and adapting effective interventions that are feasible, acceptable, and appropriate for utilization in “real-world” care settings for persons with SMI that are often characterized by limited time and fiscal resources.

Future directions for this research include additional adaptations to further enhance the fit of MIDAS components within typical RCF environments, as well as developing a better understanding of how a greater reliance on technology and remote forms of communication can facilitate MIDAS training and implementation. Refining and testing these approaches should facilitate future MIDAS dissemination endeavors in which onsite, in-person training and support may not be feasible for reasons such as health concerns that may restrict facility access (e.g., future waves of the pandemic) or within more geographically distributed settings where an onsite presence by a research team is not logistically practical.

## Declarations

### Standards of Reporting

This manuscript utilized the Strengthening the Reporting of Observational Studies in Epidemiology (STROBE) and the Consolidated Criteria for Reporting Qualitative Research (COREQ) guidelines to inform reporting.

## References

[CR1] Aarons GA, Hurlburt M, Horwitz SM (2011). Advancing a conceptual model of evidence-based practice implementation in public service sectors. Administration and Policy in Mental Health and Mental Health Services Research.

[CR2] American Diabetes Association (2004). Consensus development conference on antipsychotic drugs and obesity and diabetes. Obesity Research.

[CR3] Bartels SJ, Pratt SI, Aschbrenner KA, Barre LK, Naslund JA, Wolfe R (2015). Pragmatic replication trial of health promotion coaching for obesity in serious mental illness and maintenance of outcomes. American Journal of Psychiatry.

[CR4] Beary M, Hodgson R, Wildgust HJ (2012). A critical review of major mortality risk factors for all-cause mortality in first-episode schizophrenia: Clinical and research implications. Journal of Psychopharmacology.

[CR5] Blixen CE, Kanuch S, Perzynski AT, Thomas C, Dawson NV, Sajatovic M (2016). Barriers to self-management of serious mental illness and diabetes. American Journal of Health Behavior.

[CR6] Bobes J, Arango C, Garcia-Garcia M, Rejas J (2010). Healthy lifestyle habits and 10-year cardiovascular risk in schizophrenia spectrum disorders: An analysis of the impact of smoking tobacco in the CLAMORS schizophrenia cohort. Schizophrenia Research.

[CR7] Brown S (1997). Excess mortality of schizophrenia. A meta-analysis. The British Journal of Psychiatry.

[CR8] Buckley PF, Meyer JM (2010). Substance abuse and schizophrenia.

[CR9] Cabassa LJ, Camacho D, Vélez-Grau CM, Stefancic A (2017). Peer-based health interventions for people with serious mental illness: A systematic literature review. Journal of Psychiatric Research.

[CR10] Cabassa LJ, Stefancic A, O’Hara K, El-Bassel N, Lewis-Fernández R, Luchsinger JA (2015). Peer-led healthy lifestyle program in supportive housing: Study protocol for a randomized controlled trial. Trials.

[CR11] Casey DE, Hansen TE, Meyer J, Nasrallah H, Meyer JM, Nasrallah HA (2009). Excessive mortality and morbidity associated with schizophrenia. Medical illness and schizophrenia.

[CR12] Chesney E, Goodwin GM, Fazel S (2014). Risks of all-cause and suicide mortality in mental disorders: A meta-review. World Psychiatry.

[CR13] Chouinard V-A, Pingali SM, Chouinard G, Henderson DC, Mallya SG, Cypess AM (2016). Factors associated with overweight and obesity in schizophrenia, schizoaffective and bipolar disorders. Psychiatry Research.

[CR14] Cimo A, Stergiopoulos E, Cheng C, Bonato S, Dewa CS (2012). Effective lifestyle interventions to improve type II diabetes self-management for those with schizophrenia or schizoaffective disorder: A systematic review. BMC Psychiatry.

[CR15] Corbin J, Strauss A, Corbin J, Strauss A (2008). Strategies for qualitative data analysis. Basics of qualitative research: Techniques and procedures for developing grounded theory.

[CR16] Correll CU, Robinson DG, Schooler NR, Brunette MF, Mueser KT, Rosenheck RA (2014). Cardiometabolic risk in patients with first-episode schizophrenia spectrum disorders: Baseline results from the RAISE-ETP study. JAMA Psychiatry.

[CR17] Craig T, Lin S (1981). Death and deinstitutionalization. The American Journal of Psychiatry.

[CR18] Crump C, Winkleby MA, Sundquist K, Sundquist J (2013). Comorbidities and mortality in persons with schizophrenia: A Swedish national cohort study. American Journal of Psychiatry.

[CR100] Curran GM, Bauer M, Mittman B, Pyne JM, Stetler C (2012). Effectiveness-implementation hybrid designs: Combining elements of clinical effectiveness and implementation research to enhance public health impact. AMedical Care.

[CR19] Daumit GL, Dalcin AT, Dickerson FB, Miller ER, Evins AE, Cather C (2020). Effect of a comprehensive cardiovascular risk reduction intervention in persons with serious mental illness: A randomized clinical trial. JAMA Network Open.

[CR20] Dedoose 8.3.45. (2021). SocioCultural Research Consultants, LLC.

[CR200] Fiore, M. C., Jaén, C. R., Baker, T. B., Bailey, W. C., Benowitz, N. L., Curry, S. J., et al. (2008). *Treating tobacco use and dependence: 2008 update. Clinical practice guideline*. U.S. Department of Health and Human Services. Public Health Service. Rockville, MD

[CR21] Galling B, Roldan A, Nielsen RE, Nielsen J, Gerhard T, Carbon M (2016). Type 2 diabetes mellitus in youth exposed to antipsychotics: A systematic review and meta-analysis. JAMA Psychiatry.

[CR22] Goldman HH (1982). Mental illness and family burden: A public health perspective. Psychiatric Services.

[CR23] Gough SC, O’Donovan MC (2005). Clustering of metabolic comorbidity in schizophrenia: A genetic contribution?. Journal of Psychopharmacology.

[CR24] Greenhalgh T, Robert G, Macfarlane F, Bate P, Kyriakidou O (2004). Diffusion of innovations in service organizations: Systematic review and recommendations. The Milbank Quarterly.

[CR25] Heald A (2010). Physical health in schizophrenia: A challenge for antipsychotic therapy. European Psychiatry.

[CR26] Hennekens CH (2007). Increasing global burden of cardiovascular disease in general populations and patients with schizophrenia. The Journal of Clinical Psychiatry.

[CR27] Hert M, Schreurs V, Vancampfort D, Winkel R (2009). Metabolic syndrome in people with schizophrenia: A review. World Psychiatry.

[CR28] Hoang U, Goldacre MJ, Stewart R (2013). Avoidable mortality in people with schizophrenia or bipolar disorder in England. Acta Psychiatrica Scandinavica.

[CR29] Hong S, Lee EE, Martin AS, Soontornniyomkij B, Soontornniyomkij V, Achim CL (2016). Abnormalities in chemokine levels in schizophrenia and their clinical correlates. Schizophrenia Research.

[CR30] Ireys H, Achman L, Takyi A (2006). State regulation of residential facilities for adults with mental illnesses ((SMA) 06-4166).

[CR31] Jeste DV, Wolkowitz OM, Palmer BW (2011). Divergent trajectories of physical, cognitive, and psychosocial aging in schizophrenia. Schizophrenia Bulletin.

[CR32] Joseph J, Depp C, Martin AS, Daly RE, Glorioso DK, Palmer BW, Jeste DV (2015). Associations of high sensitivity C-reactive protein levels in schizophrenia and comparison groups. Schizophrenia Research.

[CR33] Kirkpatrick B, Messias E, Harvey PD, Fernandez-Egea E, Bowie CR (2008). Is schizophrenia a syndrome of accelerated aging?. Schizophrenia Bulletin.

[CR34] Klein KJ, Sorra JS (1996). The challenge of innovation implementation. Academy of Management Review.

[CR35] Koutsouleris N, Davatzikos C, Borgwardt S, Gaser C, Bottlender R, Frodl T (2014). Accelerated brain aging in schizophrenia and beyond: A neuroanatomical marker of psychiatric disorders. Schizophrenia Bulletin.

[CR36] Lee EE, Eyler LT, Wolkowitz OM, Martin AS, Reuter C, Kraemer H, Jeste DV (2016). Elevated plasma F2-isoprostane levels in schizophrenia. Schizophrenia Research.

[CR300] Lee PH, Macfarlane DJ, Lam TH, Stewart SM (2011). Validity of the international physical activity questionnaire short form (IPAQ-SF): A systematic review. International Journal of Behavioral Nutrition and Physical Activity.

[CR37] McGinty EE, Baller J, Azrin ST, Juliano-Bult D, Daumit GL (2016). Interventions to address medical conditions and health-risk behaviors among persons with serious mental illness: A comprehensive review. Schizophrenia Bulletin.

[CR38] McKibbin CL, Golshan S, Griver K, Kitchen K, Wykes TL (2010). A healthy lifestyle intervention for middle-aged and older schizophrenia patients with diabetes mellitus: A 6-month follow-up analysis. Schizophrenia Research.

[CR39] McKibbin CL, Patterson TL, Norman G, Patrick K, Jin H, Roesch S (2006). A lifestyle intervention for older schizophrenia patients with diabetes mellitus: A randomized controlled trial. Schizophrenia Research.

[CR40] Mitchell AJ, Vancampfort D, Sweers K, van Winkel R, Yu W, De Hert M (2013). Prevalence of metabolic syndrome and metabolic abnormalities in schizophrenia and related disorders—A systematic review and meta-analysis. Schizophrenia Bulletin.

[CR41] Olfson M, Gerhard T, Huang C, Crystal S, Stroup TS (2015). Premature mortality among adults with schizophrenia in the United States. JAMA Psychiatry.

[CR42] Palinkas LA, Aarons GA, Horwitz S, Chamberlain P, Hurlburt M, Landsverk J (2011). Mixed method designs in implementation research. Administration and Policy in Mental Health and Mental Health Services Research.

[CR43] Piatt EE, Munetz MR, Ritter C (2010). An examination of premature mortality among decedents with serious mental illness and those in the general population. Psychiatric Services (washington, d. c.).

[CR44] Proctor E, Silmere H, Raghavan R, Hovmand P, Aarons G, Bunger A (2011). Outcomes for implementation research: Conceptual distinctions, measurement challenges, and research agenda. Administration and Policy in Mental Health and Mental Health Services Research.

[CR45] Sajatovic M, Gunzler D, Einstadter D, Thomas C, McCormick R, Perzynski AT (2016). A preliminary analysis of individuals with serious mental illness and comorbid diabetes. Archives of Psychiatric Nursing.

[CR46] Solari CD, Cortes A, Henry M, Matthews N, Morris S (2014). The 2013 annual homeless assessment report (AHAR) to congress Part 2 estimates of homelessness in the United States.

[CR47] Somers JM, Moniruzzaman A, Patterson M, Currie L, Rezansoff SN, Palepu A, Fryer K (2017). A randomized trial examining housing first in congregate and scattered site formats. PLoS ONE.

[CR400] Torrey EF (1995). Surviving schizophrenia: A manual for families, consumers, and providers.

[CR48] Tsai J, Stroup TS, Rosenheck RA (2011). Housing arrangements among a national sample of adults with chronic schizophrenia living in the United States: A descriptive study. Journal of Community Psychology.

[CR49] Tsuang MT, Woolson RF (1978). Excess mortality in schizophrenia and affective disorders: Do suicides and accidental deaths solely account for this excess?. Archives of General Psychiatry.

[CR50] Vancampfort D, Sweers K, Probst M, Maurissen K, Knapen J, Minguet P, De Hert M (2011). Association of the metabolic syndrome with physical activity performance in patients with schizophrenia. Diabetes & Metabolism.

[CR51] Ward M, Druss B (2015). The epidemiology of diabetes in psychotic disorders. Lancet Psychiatry.

[CR52] Weinberger A, George T (2010). Nicotine and tobacco use in patients with schizophrenia.

[CR53] Weiner BJ, Lewis CC, Stanick C (2017). Psychometric assessment of three newly developed implementation outcome measures. Implementation Science.

[CR54] Wright SN, Kochunov P, Chiappelli J, McMahon RP, Muellerklein F, Wijtenburg SA (2014). Accelerated white matter aging in schizophrenia: Role of white matter blood perfusion. Neurobiology of Aging.

